# Assessment of Changes in Abrasive Wear Resistance of a Welded Joint of Low-Alloy Martensitic Steel Using Microabrasion Test

**DOI:** 10.3390/ma17092101

**Published:** 2024-04-29

**Authors:** Krzysztof Ligier, Jerzy Napiórkowski, Magdalena Lemecha

**Affiliations:** Faculty of Technical Sciences, University of Warmia and Mazury in Olsztyn, Ul. M. Oczapowskiego 11, 10-719 Olsztyn, Poland; jerzy.napiorkowski@uwm.edu.pl (J.N.); magdalena.lemecha@uwm.edu.pl (M.L.)

**Keywords:** wear testing, welded joint, abrasive wear, ball-cratering method

## Abstract

Martensitic low-alloy steels are widely used in machine construction. Due to their declared weldability, arc welding is most often used to join elements made of this type of steel. However, the high temperature associated with welding causes unfavourable changes in the microstructure, resulting in reduced abrasion resistance. Therefore, it is important to know the tribological properties of the welded joint. This article presents the results of a study on the abrasion wear resistance of a welded joint of an abrasion-resistant steel. This study tested a welded joint of an abrasive-resistant steel produced by the arc welding method. Wear testing of the welded joint was carried out under laboratory conditions by the ball-cratering method in the presence of abrasive slurry on the cross-section of the welded joint. Based on the test results, the change in the abrasive wear rate of the material as a function of the distance from the welded joint axis was determined. It was also found that the thermal processes accompanying welding caused structural changes that increased the wear rate index value. Adverse changes in the tribological properties of a welded material persist up to a distance of approx. 20 mm from the weld centre.

## 1. Introduction

Low-alloy martensitic steels, thanks to their workability and weldability as declared by their manufacturers and their resistance to abrasive wear, are widely used in the mining, agricultural, and transport industries for operating parts exposed to abrasive wear [1,2]. Due to the aforementioned functional properties of this steel type, machinery users often use these materials to shape machine operating parts from them, according to their needs, while usually using welding techniques. However, despite the declared weldability of these steel, adverse changes in the microstructure, caused by welding, are observed in the welding area [3,4,5,6,7], resulting in a reduction in the abrasion resistance commonly associated with hardness [8,9,10,11]. A change in the hardness of high-strength materials under the influence of welding is a widely analysed issue in the literature [3,12,13,14,15,16]. Previous studies [14,15,17] demonstrated that welding processes adversely affected resistance to abrasive wear within the weld metal material zone and the heat-affected zone through structural changes in these areas. The analysis of changes in the microstructure of materials under the influence of welding processes was carried out in detail [5,12,13,14,16,17] and concerns individual areas of the joint, from the base material (BM), through the heat-affected zone (HAZ) to various joint zones. Despite the accurate testing of the microstructure of individual zones of a welded joint, their tribological properties are no longer studied in such detail. Under laboratory conditions, methods of the type “rubber wheel-dry abrasive”, based on standards ASTM G65 [18] and GOST 23.208-79 [19], are commonly used to assess the wear resistance of welded joints [15,16,17]. Since the size of the test area using these methods can amount to several cm^2^, the testing covers multiple zones of the welded joint at the same time. Therefore, the abrasive wear resistance determined by these methods is a result of the wear resistance of individual areas of the joint. If an accurate assessment of the tribological properties of individual zones of a welded joint is required, methods should be used in which the area covered by a single test falls within one tested joint zone. A method for testing the abrasive wear rate, which satisfies this requirement, is the ball-cratering method.

The ball-cratering method is an abrasive wear test method which is widely used for the assessment of the abrasive wear rate of a wide range of construction materials, e.g., metals [20], ceramics [21], polymers [22,23], and thin coatings [24]. A characteristic property of the ball-cratering method is the formation of small wear marks, which are used to determine the abrasive wear rate for a material. This property of the ball-cratering method can be used to analyse the abrasive wear resistance of individual zones in the cross-section of a welded joint [25].

The aim of this work is to assess the suitability of the ball-cratering method for the analysis of wear resistance of a welded joint in its cross-section. This study aimed to determine changes in abrasive wear resistance of the cross-section of a welded joint of low-alloy martensitic steel, depending on the distance from the weld centre.

## 2. Materials and Methods

### 2.1. Welding Process

The joint was prepared using one of the commercially available low-alloy martensitic steels (ESTI s.r.l., Idro, Italy). The chemical composition, as declared by the manufacturer, and the strength properties of the test steel are provided in Table 1.

The 400 mm long sections were cut off from a ready-made ploughshare heat-treated by the manufacturer, with dimensions of 0.11 m × 0.08 m and a thickness of 12 mm, made from a low-alloy martensitic steel. The sections were then welded along the long side using the butt weld. The process of solid wire welding in active gas shielding (MAG 135) in the downhand position (PA) was used. A semi-automatic welding machine (KEMPPI Fastmig KMS 400 A); 1.2 mm diameter Lincoln Electric SupraMig HD wire [26] (EN ISO 14341:2020) designed for welding in the manufacture of earthmoving, agricultural, and mining machinery (typical chemical composition 0.08% C, 1.70% Mn, 0.85% Si); and C1 shielding gas (100% CO_2_) were used.

The following welding parameters were used:

- Welding current I = 230 A;
- Welding voltage U = 30 V;
- Welding speed for manual process about v = 0.35 m/min.

The heat introduced into the joint was calculated from the following formula [27]:

(1) $$Q=\frac{k\cdot U\cdot I\cdot 60}{v\cdot 1000}[\frac{kJ}{mm}]$$

where *Q* is the line energy [kJ/mm]; *U* is the voltage (*U* = 30 V); *I* is the current (*I* = 230 A); *k* is the thermal efficiency coefficient of the welding process (*k* = 0.8); and *v* is the welding speed (*v* = 350 mm/min).

The calculated heat input was equal to 0.946 kJ/mm. The manufacturer of the sheet to be welded allows sheet thicknesses up to 12 mm to be welded without preheating. Due to possible fluctuations in the welding speed, the heat input may exceed 1 kJ/mm. According to the sheet metal manufacturer’s recommendations, if the heat input is between 1.0 and 1.69 kJ/mm, it is recommended to increase the preheating temperature by 25 °C above the recommended preheating temperature. It was therefore decided to preheat the joined sheets to 50 °C. The preheating process was carried out in a muffle furnace for 60 min.

The sealing run (1) was made first, followed by fill-up passes (2, 3) and one capping (4), forming the face of the joint, made in subsequent stages of the welding process. The application of interpass cooling to 225 °C was carried out in air. The temperature was controlled using a pyrometer. The produced joint was not heat-treated.

### 2.2. Sample Preparation

**Figure 1:**
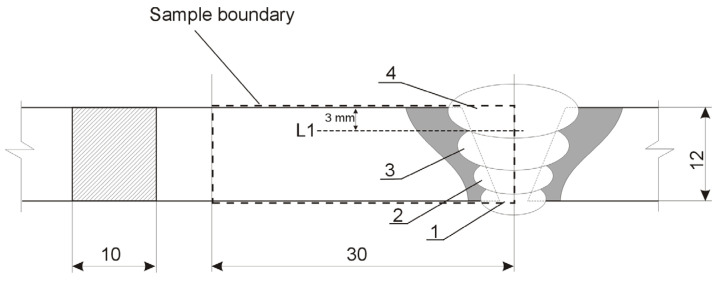
Diagram of the welded joint under test: 1—sealing run, 2, 3—fill-up passes, 4—capping, L1—hardness and wear rate test line.

The produced welded joint was cut transversely into 10 mm wide sections from which specimens were prepared for metallographic testing as well as hardness and wear rate testing. The testing of the hardness and wear rate of the produced joint was carried out at a depth of 3 mm from the top edge of the sample (L1 line—Figure 1).

The transverse surfaces were ground and polished using the Struers LaboPol-5 (Struers S.A.S., Champigny-sur-Marne, France) polisher, and the specimens for metallographic testing were additionally etched with nital (a 5% HNO_3_ alcoholic solution).

The assessment of the joint microstructure was carried out by light microscopy methods using a Keyence VHX 700 (Mechelen, Belgium) digital optical microscope.

The hardness of the welded joint was determined by the Vickers method in accordance with standard ISO 6507-1:2018 [28] using a Wilson VH1150 (London, UK) with a load of 98.1 N and a load application time of 10 s. Hardness measurements were taken every 1 mm along the L1 line (Figure 1). The hardness assessment was carried out for five specimens.

**Figure 2:**
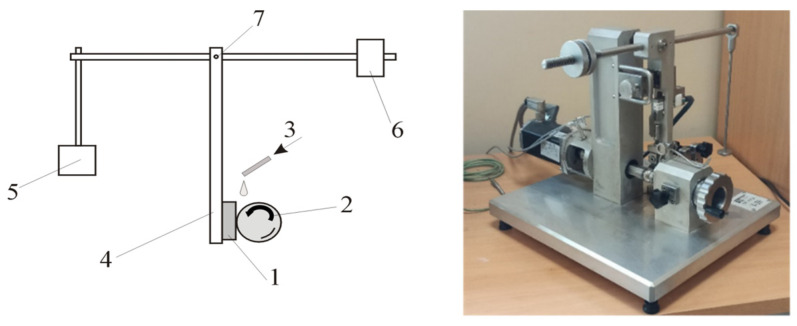
Schematic and general views of the test stand for testing the abrasive wear using the ball-cratering method. 1—sample, 2—ball (counter-sample), 3—abrasive slurry feed, 4—sample holder arm, 5—load, 6—counterweight, 7—pivot.

Abrasive wear rate testing was carried out by the ball-cratering method using a tribometer with a fixed ball system (Łukasiewicz Institute for Sustainable Technologies, Radom, Poland) (Figure 2).

A 25.4 mm (1″) diameter ball made of 100Cr6 steel, with a hardness of 58.6 HRC, was used as the counter-specimen. The abrasive wear testing was carried out according to standard EN-1071-6:2008 [27,29], using the following parameters:

- Friction assembly load: 0.4 N;
- Counter-specimen rotational speed: 150 rpm;
- Experimental run duration: 15 min;
- Sliding distance: 179.5 m.

The abrasive wear testing was carried out using an abrasive slurry prepared from aluminium oxide (Al_2_O_3_) with a grain size of 3 µm (P.P.U.H. “KOS” Stanisław Kos, Koło, Poland) and distilled water. The slurry volume concentration was approx. 2%. The slurry was fed onto the friction assembly in an amount of 1 cm^3^/min. The ball and the test surface were washed and degreased with ethyl alcohol each time.

Due to the limitations of the tribometer’s working area, the tribological test specimens covered the welded joint from the weld axis to the base material. Test runs were made every 2 mm along the line perpendicular to the joint axis, located 3 mm from the top edge of the sheet being joined (L1 line in Figure 1), with a test area length of 30 mm. The testing was carried out in six replications using six specimens.

The wear volume was determined based on the diameter of the obtained craters, measured perpendicular and parallel to the direction of movement of the abrasive particles. The crater diameters were measured using a digital optical microscope.

The wear volume was calculated using the following formula:

(2) $$V=\frac{\pi \cdot b^{4}}{64\cdot R}[{mm}^{3}]$$

where *R* is the ball radius [mm] and *b* is mean crater diameter [mm].

The wear rate index was calculated using the Archard formula [29] (EN-1071-6:2008):

(3) $$W_{r}=\frac{V}{S\cdot N}=\frac{\pi b^{4}}{64\cdot R\cdot S\cdot N}[{mm}^{3}\cdot N^{-1}\cdot m^{-1}]$$

where *W_r_* is the wear rate index [mm^3^ N^−1^ m^−1^], *R* is the ball radius [mm], *b* is the mean crater diameter [mm], *S* is the friction distance [m], and *N* is the friction assembly normal load [N].

## 3. Results and Discussion

### 3.1. Joint Microstructure

**Figure 3:**
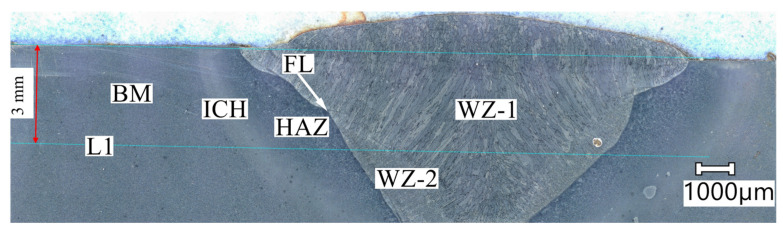
A view of the welded joint section under study: BM—base material; ICH—intercritical zone; HAZ—heat-affected zone; WZ-1 and WZ-2—weld zones; FL—fusion line; L1—testing line.

Several impacts with a concentrated heat source in the welding process resulted in noticeable changes in the microstructure in both the base material and the welded joint. In the test area, five zones with different microstructure morphologies were distinguished. These zones are marked in Figure 3.

**Figure 4:**
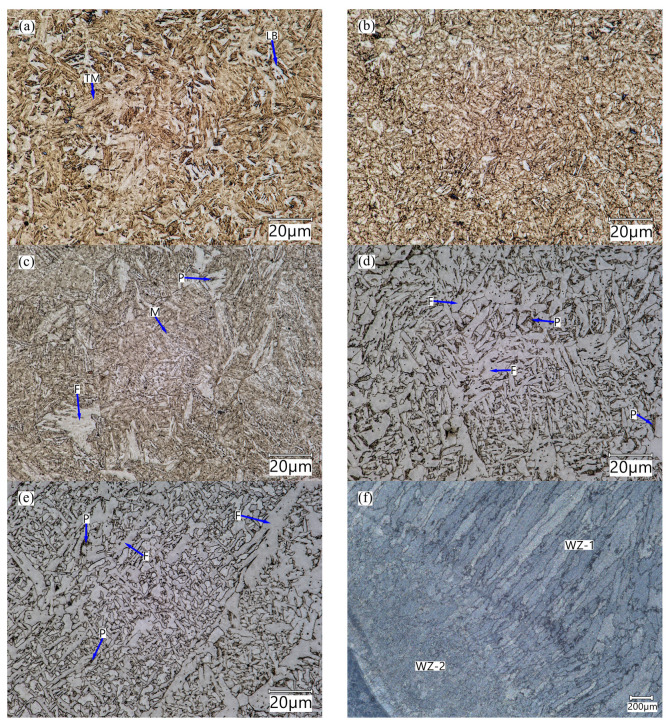
The microstructure of distinguished areas of the welded joint: (**a**) base material, (**b**) partially heat-affected zone (PHAZ), (**c**) heat-affected zone (HAZ), (**d**) weld zone within the fusion penetration zone (WZ-2), (**e**) weld filling (WZ-1), (**f**) visible differences in the microstructure orientation in zones WZ-1 and WZ-2; (TM—tempered martensite; LB—lower bainite; P—perlite; M—martensite; F—ferrite).

The base material (BM) (Figure 4a) exhibited a tempered martensite © microstructure typical of low-alloy boron steels.

Occasionally, individual grains of lower bainite (LB) can also be discerned. The heating process during welding and the rapid heat removal resulted in the transformations of the martensite microstructure (Figure 4b) in the ICH area. The intercritical heat-affected zone (ICHAZ) experiences a peak temperature between Ac1 and Ac3 and has a mixed structure of fine re-austenitized grains and tempered martensite retained from the base metal [30,31,32]. In the intercritical zone (ICH), the welded joint is characterised by the presence of tempering martensite and troostite. The observed type of structure of the intercritical zone of the welded joint is similar to that found in low-carbon low-alloy steels subjected to underhardening quenching [33]. In the HAZ microstructure, areas of tempered martensite and ferrite along with small areas of perlite were observed (Figure 4c).

The joint area, including the fill-up passes, is ferrite of varying morphology and dispersion. Within the weld zone WZ-1, structures typical of varying temperatures and cooling rates can be observed. The microstructure of this area is formed by acicular ferrite with perlite areas (Figure 4d). In the WZ-2 zone (Figure 4e), which includes the recrystallised area of the weld, acicular ferrite (F) is mainly found. In the WZ-2 area, as a result of thermal effects caused by successive transitions of the welding process, a split of the columnar microstructure into a (nearly) equiaxial (fragmented) microstructure was observed, which is typical of the annealing that occurs during multi-run welding [34]. The fragmentation of the ferritic structure results from multiple recrystallisation of the phase components of the joint and rapid heat removal. It should be noted that the clear orientation of the microstructure towards rapid heat removal is noticeable in the WZ-1 zone (Figure 4f).

### 3.2. Joint Hardness

**Figure 5:**
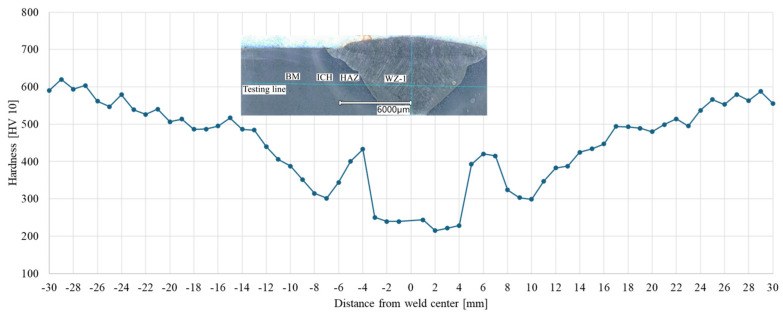
Hardness profile of one of the tested welded joint specimens.

The obtained results (Figure 5) indicate a change in the mechanical properties of the welded material, depending on the distance from the weld centre.

Within the weld area, the lowest hardness values (230 HV10) were noted in relation to the material not subjected to heat impact. The lowest hardness values were noted in WZ-1 and WZ-2 zones, which have values approx. 2.5 times lower than the hardness of the base material (approx. 600 HV 10). This should be associated with the fact that the WZ-1 and WZ-2 zones consist mainly of ferrite phases with different morphologies and small areas of perlite. The local increase in hardness near the fusion line and its subsequent decrease in the HAZ are linked to the changes in the material microstructure in this area of the joint, resulting from the welding process [34].

An increase in hardness near the fusion line results from the supercritical temperature of this area reached in the welding process and the rapid heat removal. In the heat-affected zone, pearlite and tempered martensite appear, resulting in an increase in hardness. The decrease in the hardness of the material located further away from the fusion line is due to the lower heating temperature of this area resulting in the further tempering of the martensite. This phenomenon occurs in welded joints of low-alloy steel not subjected to subsequent heat treatment [3,12,14,35].

### 3.3. Abrasive Wear Resistance

**Figure 6:**
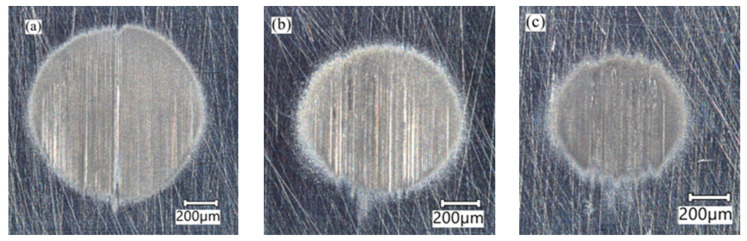
Examples of craters obtained within different zones of the test welded joint: (**a**) 1 mm distance from the joint axis—(WZ-1); (**b**) 5 mm distance from the joint axis—(HAZ); (**c**) 23 mm distance from the joint axis—(BM).

Figure 6 shows examples of craters obtained within different zones of the test welded joint.

**Figure 7:**
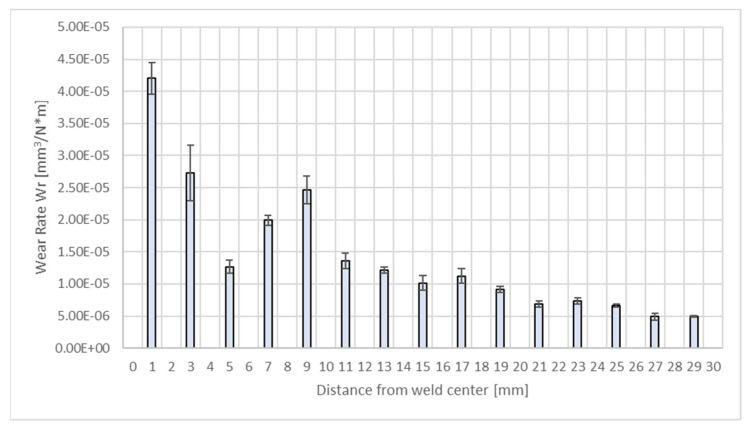
Average wear rate index values for the test welded joint, depending on the distance from the weld centre. Error bars—standard deviation.

Figure 7 shows the obtained results for the abrasive wear rate index for the welded joint, depending on the distance from the weld axis.

It can be noted that different values of the abrasive wear rate index were noted for the distinguished areas of the test welded joint. The highest abrasive wear rate occurs in the WZ-1 area located immediately near the weld axis, which is due to the low hardness of this area, resulting from the greatest proportion of welding material in the weld material as well as the greatest changes in the microstructure [34]. The WZ-2 zone, characterised by a microstructural structure similar to that of the WZ-1 zone but with grains oriented to a lesser extent, exhibits an abrasive wear rate lower than that for WZ-1. A decrease in the wear rate can be seen approx. 5 mm from the weld axis. This area is the beginning of the heat-affected zone where there is no impact of the additional material, and the wear resistance is determined by the microstructure. Due to the presence of martensite in the microstructure, the zone exhibits the greatest hardness and, thus, a lower wear rate than that in the weld area [25,36]. An increase in the abrasive wear rate is then observed at a distance of 7–9 mm from the weld centre, followed by its systematic decrease as the distance from the joint axis increases.

**Figure 8:**
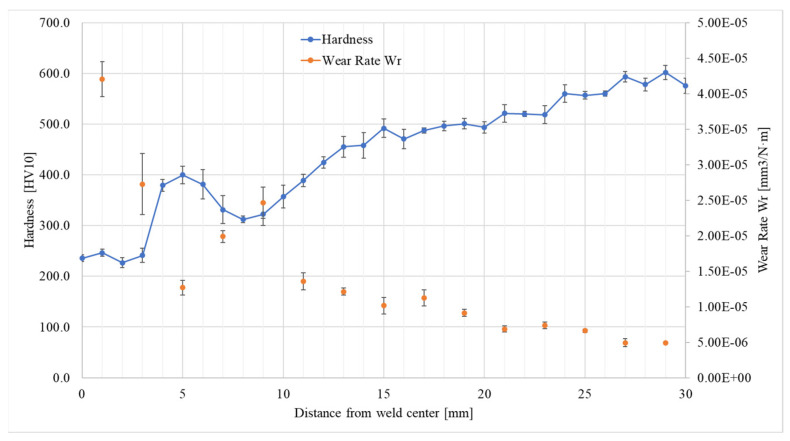
A comparison of the distribution of hardness and wear rate depending on the distance from the weld centre. Error bars—standard deviation.

When comparing the course of changes in the wear rate index and the hardness in the cross-section of the welded joint as a function of distance from the weld centre, it can be noted that there is a correlation between these quantities (Figure 8).

The increase in hardness corresponds to the reduction in wear rate, which is in line with the common view in the literature, resulting from the Archard equation that links abrasive wear resistance to the hardness of the material, which is widely presented in the literature [30,31,32,37,38,39]. The variations in the wear rate are due to the different heating temperatures of the base material during the welding process. The closer to the joint axis, the higher the tempering temperature of the martensite, which is consistent with the literature [36,40]. The authors [40] report that there is a relation between the microstructural transformation as well as the mechanical properties and the tribological response of low-alloy wear-resistant martensitic steel during sliding wear.

It is worth noting that the observed changes in the microstructure in the test area extend to approx. 10 mm (heat-affected zone boundary), while the abrasive wear rate being more than twice as high as that for the base material was noted at a distance of 17 mm from the weld centre. This indicates that the change in the tribological properties is already noticeable despite the lack of observed changes in the microstructure.

Large fluctuations in the obtained values of the wear rate and hardness index were also observed. This can be related to the accuracy of the positioning of the samples in the tribometer and the manual welding process. The accuracy of the specimen positioning overlapped with the variable weld width. Wear tests were carried out on untreated surfaces and specimen alignment was based only on the distance from the weld joint axis. The test specimens had a thickness of 10 mm, and such a distance separates the different tested joint surfaces. Due to the manual welding process, the width of the weld being produced can vary, and consequently, the boundaries of individual zones in the cross-section can be located at different distances from the weld centre. This has resulted in the fact that some test runs (despite efforts) may have been conducted in different sub-zones of the joint under test.

Three different modes of abrasive wear can be distinguished, i.e., cutting, ploughing, and wedge formation [41]. In all these modes, grooves are formed on the worn surface. However, only the cutting mode leads to material removal, i.e., wear, while the ploughing and wedge formation modes mainly lead to plastic deformation of the surface material. Therefore, the resulting abrasive wear factor will depend on the dominant wear mode in the actual abrasive contact. A high abrasive wear rate is the result of a combination of high hardness, which reduces the penetration rate of the abrasive grains, and high fracture and peel strength of the material [42].

In all the tested welded joint zones, the dominant wear process is ploughing, caused by the action of sharp-edged abrasive grains on the material surface. Ploughing is accompanied by plastic deformation with an intensity that depends on the microstructure of the material in the tested zone.

**Figure 9:**
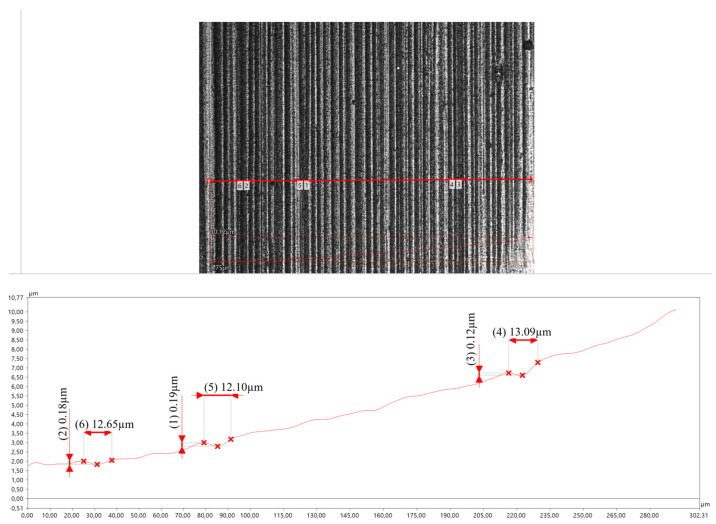
The surface of the crater made 23 mm from the axis of the welded joint.

On the surface of the crater made in the area of the base material (approx. 23 mm from the weld axis), scratches and grooves run parallel to the direction of abrasive travel along the surface (Figure 9). The grooves are narrow and shallow, and there is only occasional slight plastic deformation of the material in the form of indentations and material spreading in their area. The cutting of irregularities is the main abrasive wear type in this case [25,36].

**Figure 10:**
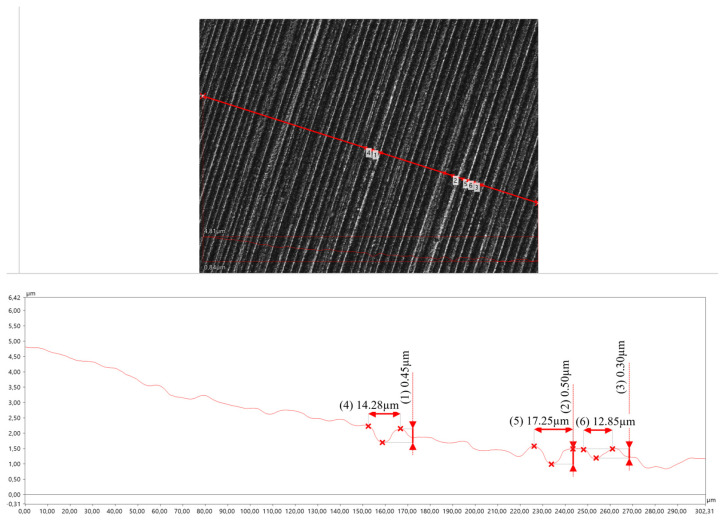
The surface of the crater made 12 mm from the axis of the welded joint.

As the test area approaches closer (remaining outside the HAZ) to the axis of the welded joint, the grooves become deeper. However, they do not lose their orientation in relation to the direction in which the abrasive moves across the surface of the specimen. The plastic deformation of the material is slightly increased due to the decreasing hardness value of the material [36] (Figure 10).

**Figure 11:**
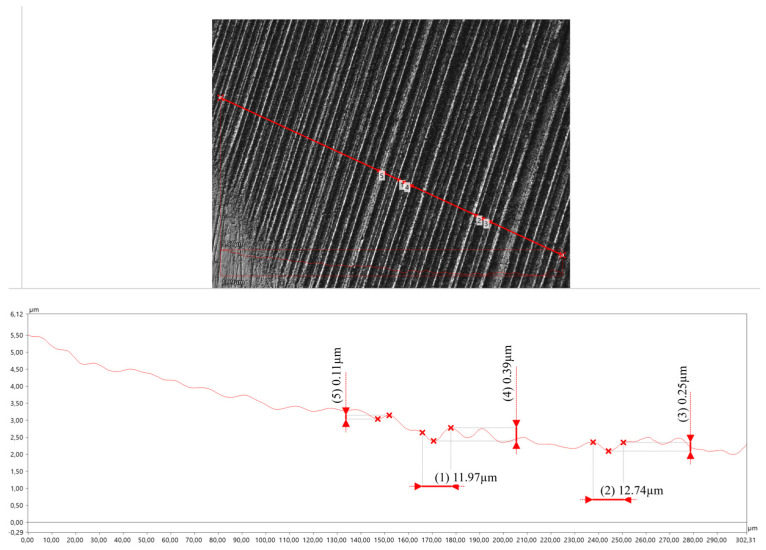
The surface of the crater made 5 mm from the axis of the welded joint.

Approximately 5 mm from the axis of the welded joint, in the heat-affected zone (HAZ), the depth of cracks and grooves decreases slightly (Figure 11). This can be explained by the increased hardness of the microstructure. Furthermore, plastic deformation (pits) is visible in the grooves, which is related to the presence of areas of ferrite in the microstructure [36].

**Figure 12:**
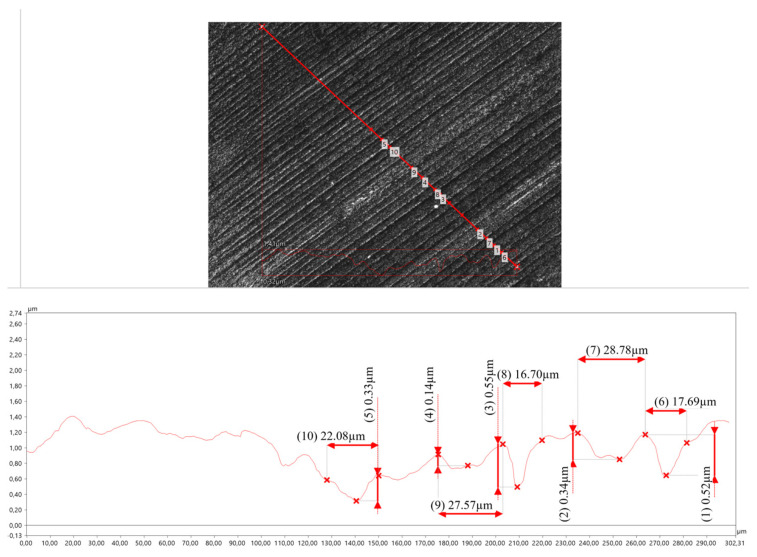
The surface of the crater made 3 mm from the axis of the welded joint.

In the WZ-1 and WZ-2 zones (0 to 4 mm from the welded joint axis), ploughing is still the dominant wear process (Figure 12).

The scratches and grooves on the specimen surface are characterised by varying depth and width. This is due to the different abrasive wear resistance of ferrite and pearlite, and thus the different abrasive wear mechanisms taking place. The perlite bands are subjected to wear by the shearing of irregularities, while the ferrite is mainly subjected to wear by furrowing with local plastic deformation of the material. The furrows are deep and pushed material is visible at their edges. The plastic deformation takes the form of sharp-edged pits and is more severe than in the HAZ. This is due to the pressing of hard abrasive grains into the relatively soft material [36].

Analysis of the surfaces of the test specimens showed that the softer microstructures are subjected to abrasive wear mainly due to furrowing. In the case of harder and more brittle structures, material loss is caused by micro-cutting, which further smooths the worn surface [36].

Welded joints of low-carbon martensitic steels with boron micro-additives, which are the subject of this study, should be heat-treated. Heat treatment of a welded joint can prolong the life of machine components exposed to abrasive wear.

## 4. Conclusions

This study’s results show that the thermal processes accompanying welding reduce the hardness of the material and increase the wear rate index value. These changes mostly affect the weld area and the heat-affected zone. The weld area of the welded joint proved to be the least resistant to abrasive wear. The wear rate index for this area proved to be greater by 5–8.5 times than that for the base material, with higher values noted closest to the weld centre. The heat-affected zone (HAZ) exhibits a variable abrasive wear rate. In the HAZ area located closest to the weld, a sudden decrease in the wear rate to a value 2.5 times higher than that for the base material was observed, followed by its subsequent increase to a value five times higher (approx. 9 mm from the weld centre) than that for the base material. Adverse changes in the tribological properties of a welded material persist up to a distance of approx. 20 mm from the weld centre.

Due to the relatively small wear marks created by the ball-cratering method, it can be applied to accurately assess the abrasion resistance of selected areas of a welded joint in its cross-section.

## Figures and Tables

**Table 1:** Declared chemical composition and strength properties of test steel.

Chemical Element	C	Si	Mn	P	S	Cr	Ni	Mo	B
Content [%]	0.28	0.35	1.40	Max. 0.30	Max. 0.03	0.50	0.30	0.25	Max. 0.004
**Declared hardness**	HB 470–530 (over the entire profile thickness after heat treatment)								
**Tensile strength Rm**	1770 MPa								
**Yield point Re**	1330 MPa								

## Data Availability

Data are contained within the article.

## References

[1] Konat Ł., Jasiński R., Białobrzeska B., Szczepański Ł. (2021). Analysis of the static and dynamic properties of wear-resistant Hardox 600 steel in the context of its application in working elements. Mater. Sci.-Pol..

[2] Białobrzeska B., Jasiński R., Konat Ł., Szczepański Ł. (2021). Analysis of the Properties of Hardox Extreme Steel and Possibilities of Its Applications in Machinery. Metals.

[3] Bramowicz M., Kulesza S., Lewalski P., Szatkowski J. (2016). Structural Studies of Welds in Wear-Resistant Steels. Acta Phys. Pol. A.

[4] Gáspár M. (2019). Effect of Welding Heat Input on Simulated HAZ Areas in S960QL High Strength Steel. Metals.

[5] Lu Y., Peer A., Abke T., Kimchi M., Zhang W. (2018). Subcritical heat affected zone softening in hot-stamped boron steel during resistance spot welding. Mater. Des..

[6] Saxena A., Kumaraswamy A., Madhu V., Madhusudhan R.G. (2018). Study of Tribological Characteristics of Multi-pass SMAW Armox 500T Steel Joints. J. Mater. Eng. Perform..

[7] Montero J., García A., Varela A., Zaragoza S., Artiaga R., Mier J.L. (2010). A study on wear of welded joins for pipelines. Weld. Int..

[8] Konat Ł., Pękalski G. (2020). “Overview of Materials Testing of Brown-Coal Mining Machines” (Years 1985–2017). Mining Machines and Earth-Moving Equipment: Problems of Design, Research and Maintenance.

[9] Adamiak M., Górka J., Kik T. (2009). Comparison of abrasion resistance of selected constructional materials. J. Achiev. Mater. Manuf. Eng..

[10] Bhakat A.K., Mishra A.K., Mishra N.S. (2007). Characterization of wear and metallurgical properties for development of agricultural grade steel suitable in specific soil conditions. Wear.

[11] Białobrzeska B. (2022). The influence of boron on the resistance to abrasion of quenched low-alloy steels. Wear.

[12] Konat Ł., Białobrzeska B., Białek P. (2017). Effect of Welding Process on Microstructural and Mechanical Characteristics of Hardox 600 Steel. Metals.

[13] Brykov M.N., Petryshynets I., Džupon M., Kalinin Y.A., Efremenko V.G., Makarenko N.A., Pimenov D.Y., Kovác F. (2020). Microstructure and Properties of Heat Affected Zone in High-Carbon Steel after Welding with Fast Cooling in Water. Materials.

[14] Wan Z., Guo W., Jia Q., Xu L., Peng P. (2018). Hardness Evolution and High Temperature Mechanical Properties of Laser Welded DP980 Steel Joints. High Temp. Mater. Proc..

[15] Górka J. (2020). The assessment of the quality of welded joints made of abrasion-resistant plates using the nanocrystalline filler metal. J. Min. Metall. Sect. B Metall..

[16] Konat Ł., Białobrzeska B. (2022). Effect of Welding Technique and Thermal Heatment Parameters on Abrasive Wear of Steel S355. Tribologia.

[17] Tomków J., Czupryński A., Fydrych D. (2020). The Abrasive Wear Resistance of Coatings Manufactured on High-Strength Low-Alloy (HSLA) Offshore Steel in Wet Welding Conditions. Coatings.

[18] (2021). Standard Test Method for Measuring Abrasion Using the Dry Sand/Rubber Wheel Apparatus.

[19] (1979). Ensuring of Wear Resistance of Products. Wear Resistance Testing of Materials by Friction against Loosely Fixed Abrasive Particles.

[20] Marques F., da Silva W.M., Pardal J.M., Tavares S.S.M., Scandian C. (2011). Influence of Heat Treatments on the Micro-Abrasion Wear Resistance of a Superduplex Stainless Steel. Wear.

[21] Antunes P.V., Ramalho A. (2003). Study of Abrasive Resistance of Composites for Dental Restoration by Ball-Cratering. Wear.

[22] Farfán-Cabrera L.I., Gallardo-Hernández E.A., de la Rosa C.S., Vite-Torres M. (2017). Micro-Scale Abrasive Wear of Some Sealing Elastomers. Wear.

[23] Ligier K., Olejniczak K., Napiórkowski J. (2021). Wear of polyethylene and polyurethane elastomers used for components working in natural abrasive environments. Polym. Test..

[24] Silva F., Martinho R., Baptista A. (2014). Characterization of Laboratory and Industrial CrN/CrCN/Diamond-Like Carbon Coatings. Thin Solid Film..

[25] Ligier K., Bramowicz M., Kulesza S., Lemecha M., Pszczółkowski B. (2023). Use of the Ball-Cratering Method to Assess the Wear Resistance of a Welded Joint of XAR400 Steel. Materials.

[26] (2020). Welding Consumables Wire Electrodes and Weld Deposits for GAS shielded Metal Arc Welding of Non Alloy and Fine Grain Steels. Classification.

[27] (2017). Specification and Qualification of Welding Procedures for Metallic Materials Welding Procedure Test.

[28] (2018). Metallic Materials Vickers Hardness Test.

[29] (2008). Advanced Technical Ceramics—Methods of Test for Ceramic Coatings—Part 6: Determination of the Abrasion Resistance of Coatings by a Micro-Abrasion Wear Test.

[30] Francis J.A., Mazur W., Bhadeshia H.K.D.H. (2006). Review type IV cracking in ferritic power plant steels. Mater. Sci. Technol..

[31] David S.A., Siefert J.A., Feng Z. (2013). Welding and weldability of candidate ferritic alloys for future advanced ultrasupercritical fossil power plants. Sci. Technol. Weld. Join..

[32] Wang Y., Kannan R., Li L. (2018). Correlation between intercritical heat-affected zone and type IV creep damage zone in grade 91 steel. Metall. Mater. Trans. A.

[33] Konat Ł., Zemlik M., Jasiński R., Grygier D. (2021). Austenite grain growth analysis in a welded joint of high-strength martensitic abrasion-resistant steel hardox 450. Materials.

[34] Konat Ł. (2021). Technological, microstructural and strength aspects of welding and post-weld heat treatment of martensitic, wear-resistant Hardox 600 steel. Materials.

[35] Di H., Sun Q., Wang X., Li J. (2017). Microstructure and properties in dissimilar/similar weld joints between DP780 and DP980 steels processed by fiber laser welding. J. Mater. Sci. Technol..

[36] Łętkowska B., Dudziński W., Frydman S. (2012). Abrasive wear for selected grades of low-carbon boron steels at different states of heat treatment. Q. Tribol..

[37] Liu Y., Liskiewicz T.W., Beake B.D. (2019). Dynamic changes of mechanical properties induced by friction in the Archard wear model. Wear.

[38] Hsu S.M., Shen M.C., Ruff A.W. (1997). Wear prediction for metals. Tribol. Int..

[39] Napiórkowski J., Lemecha M., Konat Ł. (2019). Forecasting the Wear of Operating Parts in an Abrasive Soil Mass Using the Holm-Archard Model. Materials.

[40] Li C., Deng X., Huang L., Jia Y., Wang Z. (2020). Effect of temperature on microstructure, properties and sliding wear behavior of low alloy wear-resistant martensitic steel. Wear.

[41] Hokkirigawa K., Kato K. (1988). An experimental and theoretical investigation of ploughing, cutting and wedge formation during abrasive wear. Tribol. Int..

[42] Rendón J., Olsson M. (2009). Abrasive wear resistance of some commercial abrasion resistant steels evaluated by laboratory test methods. Wear.

